# Polyphenolic spectrum of cornelian cherry fruits and their health-promoting effect

**DOI:** 10.1515/biol-2025-1117

**Published:** 2025-06-17

**Authors:** Tunde Jurikova, Nela Skowronkova, Magdalena Zvonkova, Jana Orsavova, Sezai Ercisli, Libor Dokoupil, Katarina Fatrcova-Sramkova, Anna Adamkova, Jiri Mlcek

**Affiliations:** Institute for Teacher Training, Faculty of Central European Studies, Constantine the Philosopher University in Nitra, Nitra, 94974, Slovakia; Department of Food Analysis and Chemistry, Faculty of Technology, Tomas Bata University in Zlin, Vavreckova 5669, Zlin, 760 01, Czech Republic; Language Centre, Tomas Bata University in Zlín, Štefánikova 5670, Zlín, 760 01, Czech Republic; Department of Horticulture, Faculty of Agriculture, Ataturk University, Erzurum, 25240, Turkey; Department of Breeding and Propagation of Horticultural Plants, Faculty of Horticulture, Mendel University in Brno, Valtická 337, Lednice, 691 44, Czech Republic; Institute of Nutrition and Genomics, Faculty of Agrobiology and Food Resources, Slovak University of Agriculture in Nitra, Tr. A. Hlinku 2, Nitra, 94976, Slovak Republic

**Keywords:** *Cornus mas*, polyphenols, anti-inflammatory, metabolic, antidiabetic, antimicrobial, anticancer effect

## Abstract

Although cornelian cherry is an underutilized fruit species, its fruits have a high biological value due to valuable biologically active substances, especially polyphenols. The total content of polyphenols accounts for 37% of all the bioactive substances examined. Flavonols, anthocyanins, flavan-3-ols, and phenolic acids represent the main groups of phenolic compounds present and thanks to these compounds, cornelian cherry fruits possess mainly antioxidant, anti-inflammatory, antimicrobial, metabolic, antidiabetic, cardioprotective, and anticancer effects. This review summarizes new research aimed at popularizing this lesser-known species not only for direct consumption but also for further processing.

## 1 Introduction

Among 65 species belonging to the genus *Cornus*, only two, *Cornus mas* L. and *Cornus officinalis* Sieb. et Zucc. (*Cornaceae*), have been traditionally used since ancient times. *C. mas* (cornelian cherry) is native to southern Europe and southwest Asia, whereas *C. officinalis* (Asiatic dogwood, cornel dogwood) is a deciduous tree found in eastern Asia, primarily in China, as well as Korea and Japan [1,2]. *C. mas* L. (cornelian cherry) has been popular in garden cultivation for 4,000 years [2]. Although the fruit is one of the lesser-known and valued species, an interest in growing this crop has been increasing [3,4].

Oval or pear-shaped fruits of *C. mas* L. ranging in color from red to purple [5], represented a valuable source of bioactive substances, including polyphenols (anthocyanins, phenolic acids, flavonoids) [6,7], catechins (CATs) and tannins (37.36%), monoterpenes (26.3%), organic acids (25.9%), vitamin C (10.7%), and lipophilic compounds such as carotenoids [8,9].

90% of the polyphenolic extract obtained from cornelian cherry fruit was represented by loganoic acid, cornuside, and anthocyanins – cyanidin 3-galactoside and pelargonidin 3-galactoside [10,11]. Despite its high biological value, the fruit is classified as underutilized and forgotten fruit species [12].

Among the group of polyphenols are the most abundant phenolic acids (i.e., benzoic acid derivatives and cinnamic acid derivatives) and second flavonols, especially quercetin derivatives [13]. Irridois represent also valuable part of fruits – especially loganoic acid and cornuside [2,14]. To sum up all bioactive compounds, Przybylska et al. [15] highlighted and determined 37 bioactive compounds, which included various gallotannins (11), monomeric ellagitannins (7), dimeric ellagitannins (10), and trimeric ellagitannins (7).

The fruits of cornelian cherry have been valued in folk medicine for years [2].

The fruits can be consumed fresh or dried as a decoction [16] and they are also widely used in the food industry to produce various beverages, syrups, gels, jams, and compotes [17,18] and for the liquor and wine as well [10,19]. Farmers from Bosnia and Herzegovina produce a special alcoholic beverage “rakija” [12]. The fruits are also valuable in folk medicine to prevent and treat diarrhea, hemorrhoids, diabetes, sore throat, indigestion, measles, chickenpox, anemia, rickets, liver, and kidney diseases [20].

Furthermore, they can be used as a flavoring ingredient in functional ice creams, desserts, and cakes [21,22].

In general, studies about biologically active compounds in cornelian cherry have been published, but only a few of them have focused on the main group of bioactive substances – polyphenolic compounds, which are responsible for their antioxidant activity (AA) and health benefits. Moreover, the review discusses the influence of locality, climatic conditions, and chemical extraction methodology. Therefore, the main aim of the present review is to provide an overview of the main polyphenolic compounds contained in fruits including their health benefits.

Databases WOS, Scopus, and PubMed were used to summarize information for this review.

## 2 Total polyphenol content (TPC) of *C. mas* fruit

According to De Biaggi et al. [5], the TPC reached up to 37% of all bioactive compounds presented (polyphenols, monoterpenes, organic acids, and vitamin C) in fruit fresh weight (FW). Therefore, it is very important to focus on this group of compound, particularly with regard to its variability.

The polyphenol content exhibited wide variability despite the identical cultivation origin. For example, the TPC of *C. mas* fruit from Ukraine determined by the Folin–Ciocalteu reagent by Klymenko et al. [2] showed a wide range of values from 100.71 (“Koralovyi”) to 924.65 mg GAE·100 g^−1^ FW (“Uholok”). On the other hand, in a comparative study of the interspecific hybrids *C. mas* × *C. officinalis* contained higher concentration of polyphenolic compounds than *C. officinalis* and *C. mas*. In another study provided by Klymenko et al. [23], 20 different cultivars collected in the M.M. Gryshko National Botanical Garden of NAS of Ukraine were analyzed for TPC. The study found that TPC was genotype dependent, with values ranging from 91.34 (“Kozerog”) to 289.79 (“Bolgarskyj”) mg GAE·100 g^−1^ FW. These results demonstrated that TPC is dependent on cultivar and genotype.

Six genotypes of cornelian cherry, selected from spontaneous flora in different areas in Romania, were studied for TPC as well. Cosmulescu et al. [24] reported TPC ranging from 163.69 (S1) to 359.28 (H2) mg GAE·100 g^−1^ FW. Genotypes H2 and H3 had the highest TPC (359.28 and 343.50 mg GAE·100 g^−1^ FW, respectively) [24]. Skender et al. [12] studied the TPC of 22 promising local cornelian cherry (*C. mas* L.) genotypes from Bosnia and Herzegovina. The results showed that the TPC was quite variable among studied genotypes and ranged from 1,240 to 6,958 mg GAE·100 g^−1^ FW. They found statistically significant differences among genotypes in terms of bioactive compounds content at the 0.05 level of significance. Additionally, Kucharska et al. [25] investigated cultivars of Polish breeding in relation to fruit morphological parameters. According to their findings, the Szafer cultivar was the richest source of polyphenols (464 mg·100 g^−1^ FW), with a fruit length of 1.72 cm (second longest) and displayed the highest fruit mass max (4.41 g). In contrast, the cultivar Juliusz had the lowest polyphenol content (262 mg GAE·100 g^−1^ FW), the smallest fruit length (1.10 cm), and the lowest fruit mass 3.24 g.

Table 1 shows that the polyphenol content in cornelian cherry fruit is influenced by genetic factors as well as the fruit’s origin and cultivation environment [26]. The TPC in *C. mas* of Greek cultivars was 1 from 592 mg GAE; in cultivars from Azerbaijan 1,097–2,695 mg GAE 100 g^–1^ DW, and in Turkish cultivars, 2,659–7,483 mg GAE 100 g^–1^ DW [26,27].

**Table 1:** TPC in relation to place of cultivation of *C. mas* fruit samples (mg GAE·100 g^−1^ FW/DW)

Place of cultivation	TPC (mg GAE·100 g^−1^ FW)	Source
Ukraine	100.71–924.65 FW	Klymenko et al. [2]
	91.34–289.79 FW	Klymenko et al. [23]
Czech Republic	61–253 FW	Cetkovská et al. [27]
	261–811 FW	Rop et al. [17]
Bosnia and Herzegovina	1240–6958 FW	Skender et al. [12]
	38.98–103.37 FW	Drkenda et al. [33]
Poland	262–464 FW	Kucharska et al. [25]
Turkey	653–1009 FW	Sengul et al. [32]
Serbia	47.60–116.38 FW	Bijelić et al. [28]
Montenegro	47.60–116.38 FW	Martinović and Cavoski [30]
	32.54–157.06 FW	Jaćimović et al. [31]

Cultivars cultivated in the Czech Republic displayed TPC content between 61 and 253 mg GAE·100 g^−1^ FW. Based on TPC and AA, Ekotišnovský, Fruchtal, and Ruzyňský cultivars were recommended for breeding programs [17]. Rop et al. [17] reported a significant difference between the Czech cultivar Devin, which had the lowest TPC at 261 mg GAE·100 g^−1^ FW, and the Ukranian cultivar Vydubieckii, which exhibited the highest TPC (811 mg GAE·100 g^−1^ FW).

From the territory of Serbia, Bijelić et al. [28] reported 47.60–116.38 mg GAE·100 g^−1^ FW and Milenković-Andjelković et al. [29] 9.89 ± 0.45 to 117.34 ± 1.40 mg of gallic acid equivalents GAE·100 g^−1^ extract dry matter DW. According to experiment of Martinović and Cavoski the content of TPC was reported 47.60–116.38 mg GAE·100 g^−1^ fresh weight FW for samples from Jaćimović et al. [31] reported 32.54–157.06 mg GAE·100 g^−1^ FW). Similarly, Sengul et al. [32] examined five different varieties from Turkey and determined TPC ranging from 6.53 to 10.09 μg GAE mg^−1^ FW. Drkenda et al. [33] reported TPC in cornelian cherry samples from Bosnia and Herzegovina ranging from 38.98 to 103.37 mg GAE·100 g^−1^ FW.

Total yield efficiency was negatively correlated with TPC, as demonstrated by Gunduz et al. [35]. TPC is also associated with fruit color, which is linked to anthocyanin content [24,28]. The above-mentioned variety “Szafer” with the most intense purple color thus showed the highest TPC of all varieties tested [25,34].

TPC, which correlates with anthocyanin content (and thus fruit color), is also influenced by the fruit’s maturity stage, as shown in a study by Gunduz et al. [35]. In the early stages of ripeness, yellow-colored fruits showed the lowest concentration of polyphenols (4.162 mg GAE·100 g^−1^ DW), while at the end of ripeness, red fruits contained the highest concentration of polyphenols (8.206 mg GAE·100 g^−1^ DW).

Dupak et al. [36] brought attention to the fact that polyphenolic compounds vary in different parts of the fruit. TPC in the pulp showed significantly (*p* < 0.001) lower concentration compared to the stone. The value of TPC in the stone was 29.608 mg GAE·100 g^−1^ DW, while the pulp contained only 10.204 mg GAE·100 g^−1^ DW.

Polyphenolic content is also affected by storage conditions. Moldovan et al. [37] observed the effect of storage temperature on the stability of polyphenolic compounds in cornelian cherry (*C. mas* L) in the dark at four different temperatures – 2, 22, 55, and 75°C. The results showed that the polyphenolic substances stored at 75°C had the lowest stability. According to the results of the experiments, they recommended storage for at least 2 months at room temperature, when no significant loss of bioactive substances, including polyphenols, was observed.

Tarko et al. [38] studied the effect of the extraction method on TPC. The results of the experiment showed that the best result was obtained by 80% methanol solution – TPC was 16.36 ± 0.37 mg·g^−1^ FW compared to 10.33 ± 0.38 mg·g^−1^ FW in 80% ethanol solution, 4.63 ± 0.47 mg·g^−1^ FW in aqueous solution and 0.07 ± 0.00 mg·g^−1^ FW in methylene chloride solution. Szczepaniak et al. [39] compared the TPC in ethanol and aqueous extracts of *C. mas* fruits. The observed values ranged from 25.98 ± 4.76 to 5.50 ± 0.66 mg GAE·g^−1^ DW and 7.75 ± 0.13 to 2.90 ± 0.01 mg GAE·g^−1^ DW, respectively.

Processing also has a significant impact on TPC. Tarko et al. [40] demonstrated that the differences in TPC in fresh fruit were 6.11 ± 0.08 mg·g^−1^ FW CAT equivalent, 0.75 ± 0.12 mg·ml^−1^ CAT equivalent in CM in wine and 1.54 ± 0.15 mg·ml^−1^ CAT equivalent in CM (*C. mas*) liqueur. De Biaggii et al. [5] presented four major polyphenolic compounds such as ellagic acid, epicatechin, CAT, and chlorogenic acid.

According to Milenković-Andjelković et al. [29], TPC is also influenced by the year of cultivation, which is shaped by climatic conditions of growing season [17].

## 3 Polyphenolic spectrum of *C. mas* fruit

The main groups of phenolic compounds found in fruits include flavonols, anthocyanins, flavan-3-ols, and phenolic acids [29].

The total phenolic content (359.28 and 343.50 mg GAE 100 g^−1^ FW, respectively) and flavonoid content of cornelian cherry represented (54.26 and 64.48 mg QE 100 g^−1^ FW, respectively) with antioxidant capacity (2.39 and 2.71 mmol Trolox 100 g^−1^ FW, respectively) were genotype and cultivar dependent [24].

The fresh berries from the group of polyphenols were predominated – especially ellagic acid, epicatechin, CAT, and chlorogenic acid. Cinnamic acid derivatives – ferulic acid, coumaric acid, and caffeic acid – were represented in smaller amounts [5]. Moldovan et al. [41] determined quercetin-3-*O* glucuronide (471 mg·100 g^−1^ body weight [BW]), kaempferol-3-*O*-galactoside (366.88 mg·100 g^−1^ BW), and ellagic acid as the most abundant polyphenolic compounds.

### 3.1 Phenolic acids

Different authors have determined different amounts of phenolic acids in cornelian cherry, ranging from 2.81 to 5.79 mg·g^−1^ ml in methanol extract FW [42] to 29.76–74.83 mg GAE·g^−1^ (gallic acid equivalent) DW [26].

Cosmulescu et al. [24] identified and quantified 10 phenolic acids (gallic, vanillic, chlorogenic, caffeic, syringic, *p*-coumaric, ferulic, sinapic, salicylic, ellagic, and *trans*-cinnamic). Ellagic acid exhibited the highest concentration among phenolic acids in amount 2.59 mg·100 g^−1^ FW (*C. mas*), which represented a significantly lower content compared to the highest source of ellagic acid studied (*Hippophae rhamnoides*) with a value of 15.14 mg·100 g^−1^ FW. The analysis also revealed high concentrations of caffeic acid in *C. mas* fruits (1.26 mg·100 g^−1^ FW), followed by *Prunus padus* (0.99 mg·100 g^−1^ FW). The less abundant cinnamic acid derivatives were represented by ferulic, coumaric, caffeic, and vescalaginic acids (about 2, 4, 1, and 5 mg·100 g^−1^ FW, respectively) [5].

When comparing the amount of phenolic acids in pulp and pit, a significant difference was established. The pulp contained significantly lower values (*p* < 0.001) compared to the pit. Cornelian cherry pulp had 6.659 mg·CAE g^−1^, whereas pit only had 0.621 mg CAE g^−1^ FW [36].

The structure of phenolic acids included gallic acid [24,43], ellagic acid [24,37,44], chlorogenic acid [33,45], neochlorogenic acid [46], 3-*O*-caffeoylquinic acid [44,47], *p*-coumaric acid [24], caffeic acid [24], protocatechuic acid [48], cinnamic acid [24,48], ferulic acid [48,24], sinapic acid [24], salicylic acid [24,49], syringic acid [24], vanillic acid [24,49], and rosmarinic acid [3,48].

Dzydzan et al. [47] demonstrated that the amount of phenolic acids is related to the variety and color of the fruit. They determined a total phenolic acid content of 21.57 mg·g^−1^ DW for yellow CM fruits and 12.86 mg·g^−1^ DW for red CM fruits.

According to Sochor et al. [45], “Devin,” “Vydubeckij,” and “Titus” were the most valuable source of chlorogenic acid (135.6, 110.9, and 115.1 mg 100 g^–1^ FW, respectively).

### 3.2 Flavonoids

In a recent study by Cosmulescu et al. [24], the total flavonoid content found in *C. mas* fruits was 17.27 mg QE·100 g^−1^ FW (quercetin equivalent). Compared to another less explored species, the order of total flavonoid content was as follows*: P. padus > H. rhamnoides > Prunus spinosa > R. fruticosus* > *R. canina* > *C. monogyna* > *C. mas*. When comparing the species, the total flavonoid content of *P. padus* was found to be higher compared to the other species, e.g., 9.58 times higher than the content recorded in *C. mas*.

Stanković et al. [50] evaluated the total flavonoid content of *C. mas* fruits from the Pčinja River Gorge area in southern Serbia. They found out the influence of different extraction procedures. Water extracts of *C. mas* fruits contained 3.53 mg·g^−1^ of flavonoids, while ethyl acetate extracts yielded a higher concentration of 41.49 mg·g^−1^ FW.

Flavonoid content is related to the timing of fruit collection, different content was investigated in early, mid, and late-ripening cultivars [17]. Levon and Klymenko [51] evaluated *C. mas* varieties grown in Ukraine in terms of total flavonoid content with respect to ripening time. The results of the experiment showed that among the early-ripening *C. mas* cultivars, the most promising were the cultivars “Pervenets” and “Volodimirskii,” among the mid-ripening *C. mas* cultivars the cultivars “Shajdarovoi,” “Vydubetskyi,” and “Titus,” and among the late-ripening *C. mas* cultivars the cultivar “Sokoline.” The flavonol content of the fruits of the early-ripening *C. mas* cultivars was 34–176 mg·l^−1^, the medium-ripening cultivars in the range 27–165 mg·100 g^−1^ DW, and the late-ripening cultivars in the range 36–88 mg·100 g^−1^ DW.

In summary, the most abundant flavonoids were quercetin [45], quercetin 3-*O*-robinobioside [33], quercetin 3-*O*-glucuronide [52,47], quercetin-3-*O*-xyloside [33], quercetin-3-*O*-rhamnoside [33,45], quercetin-3-*O*-glucoside [48,53], quercetin-3-*O*-galactoside [33,53,54], kaempferol 3-*O*-galactoside [53], kaempferol 3-glucoside [54,33,29], myricetin [24], myricetin 3-galactoside [48], and aromadendrin [3,53].

**Figure 1:**
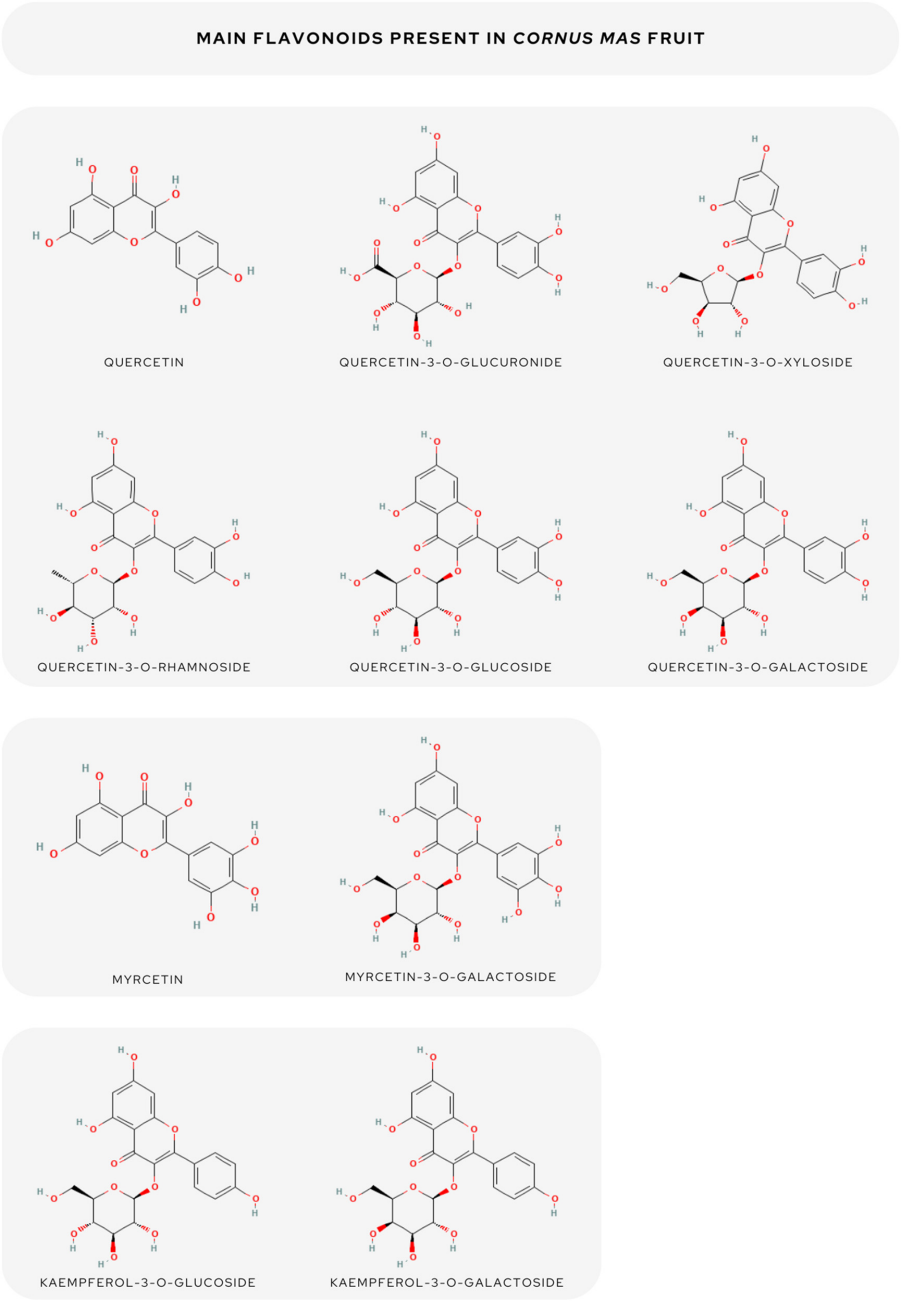
The overview of the main flavonoids of *C. mas* fruit.

The following compounds predominated among flavonols: procyanidin B1 [33,54], procyanidin B2 [33], (+) CAT [29], and (−) epicatechin [37]. Using the high-performance thin-layer chromatography method, Moldovan et al. [41] reported the average amount of flavanols to be 0.67 mg g^–1^ FW. The flavonoid content was higher in the pulp than in the pit and anthocyanins were found only in the pulp [36]. All predominated flavonoids are summed up in Figure 1.

The total flavonoid content was dependent on the extraction method. Karaaslan et al. [55] demonstrated that the acetone extract of CM fruits has the highest values of total flavonoid content. Mesgari Abbasi et al. [56] found that the mean total flavonoids are 0.07 ± 0.01% DW in 70% methanol extract of CM fruit.

According to Sochor et al. [45], “Devin,” “Vydubeckij,” and “Titus” cultivars were the most valuable source of quercetin (24.9, 25.2, and 24.2 mg·100 g^−1^ FW, respectively).

### 3.3 Anthocyanins

Total anthocyanin content in cornelian cherry has been estimated by several authors in the range of 810–2,920 mg·kg^−1^ FW [25,28,29,57].

The total anthocyanin content in different varieties of *C. mas* fruits from the National Botanical Garden of M.M. Gryshko in Ukraine ranged from 477.1 to 850.0 mg% in the skin and 7.8–190.6 mg% in the flesh FW [58]. Similarly, Milenković-Andjelković et al. [29] determined the total anthocyanin content in *C. mas* fruits from the Vlasina region (Serbia) by high-performance liquid chromatography with 1,383.2 mg·kg^−1^ FW.

Anthocyanin content varies among different fruit parts. The anthocyanin levels determined in the pulp represented 0.54 g from 2 g of total polyphenols in cornelian cherry [36].

Levon and Klymenko [51] observed that anthocyanin content is related to ripening time. The content of total anthocyanins in *C. mas* fruits with early ripening period ranged from 9 up to 259 mg·100 g^−1^ DW, with middle ripening period from 10 to 166 mg·100 g^−1^ DW, and with late ripening period from 33 to 96 mg·100 g^−1^ DW. They found a very strong correlation between the anthocyanin and flavonol content of *C. mas* plant fruits with the early ripening period of the fruits (*r* = 0.955).

Anthocyanin content also depends on the genotype. Skender et al. [12] studied the TPC in 22 local genotypes of cornelian cherry (*C. mas* L.) from Bosnia and Herzegovina. The results demonstrated that anthocyanin content was highly variable among genotypes and ranged from 5.57 to 205.6 mg cyanide-3-glucoside equivalents 100 g^–1^ FW, respectively. Similarly, Sengul et al. [32] analyzed five different genotypes of cornelian cherry grown in Turkey. The genotypes differed significantly in total anthocyanin content. The highest total anthocyanin content was recorded in red genotype 1 (342 mg·100 ml), while yellow genotypes 2, 3, 4, and 5 reached 276, 271, 239, and 262 mg·100 ml^−1^. The influence of cultivar on anthocyanin content was reported by Kucharska et al. [8]. According to the study, the “Czarny” variety exhibited the highest amount of anthocyanin of all varieties tested (341.18 mg 100 g^−1^ w/w). On the other hand, the “Jantarnyj variety” contained no detectable concentration of anthocyanin.

Moreover, anthocyanin content is influenced by origin. In samples from Turkey, anthocyanin content ranged from 0.12 to 4.2 mg·g^−1^ [42,26], while in Serbia, the values ranged from 0.36 to 1.27 mg·g^−1^ [28]. In the Czech Republic, the range was from 0.061 ± 0.007 to 0.347 ± 0.004 mg·g^−1^ FW [27], and in Poland, values ranging from 0.05 to 3.42 mg·g^−1^ FW were reported [8].

Anthocyanin content depends on the chemical composition of the fruit, particularly the organic acids. The differences in anthocyanin and organic acid concentrations between the tested varieties and genotypes were related to pH. High temperatures have a negative effect on the anthocyanin degradation process. The degradation rate of anthocyanin isolated from CM extracts was 1.8 times faster at 2°C, while the process was 172 times faster at 75°C [59].

The prevalent anthocyanidins present in cornelian cherry fruits were determined as follows: 3-*O*-galactosides of delphinidin (162 mg·100 g^−1^), cyanidin (166 mg·100 g^−1^), and pelargonidin; cyanidin-3-*O*-galactoside (3.82 mg 100 g^−1^ FW); as well as pelargonidin 3-*O*-glucoside (58.62 mg·100 g^−1^ FW) and 3-*O*-rutinoside (33.8 mg·100 g^−1^ FW) [1,8,41].

All identified anthocyanidins in the fruits of cornelian cherry were cyanidin 3-*O*-glucoside [6,48], cyanidin 3-*O*-robinobioside [8,47,60], cyanidin 3-*O*-rutinoside [32,42,57], pelargonidin 3-*O*-galactoside [8,61,62,63,64] pelargonidin 3-*O*-glucoside [6,42,48], pelargonidin 3-*O*-robinobioside [8,47,48], pelargonidin 3-*O*-rutinoside [53], peonidin-3-*O*-glucoside [32,33], delphinidin-3-*O*-galactoside [8,61], delphinidin 3-*O*-β-glucoside [6], and petunidin 3-glucoside [48].

**Figure 2:**
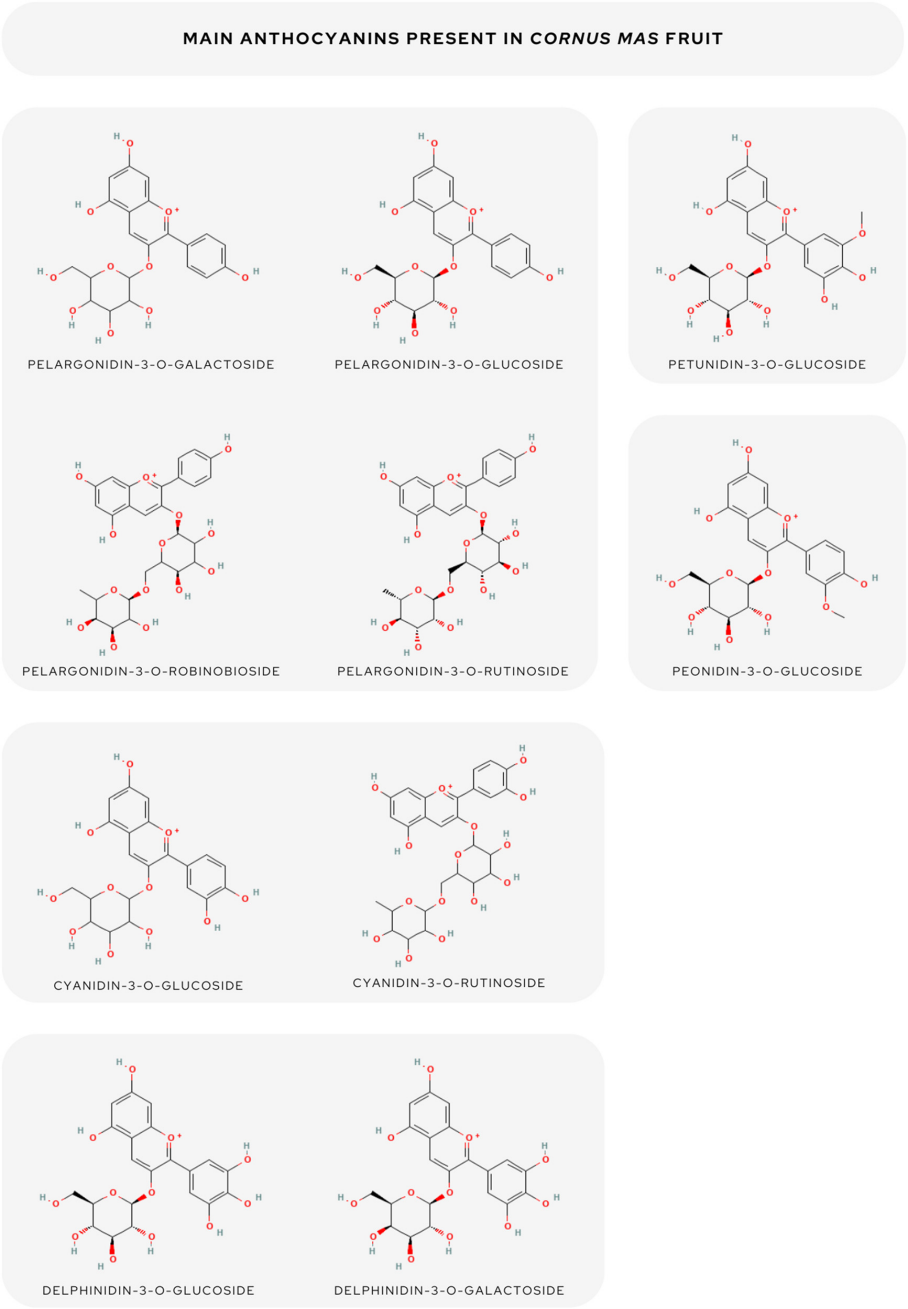
The overview of the main anthocyanidins in *C. mas* fruit.

The overview of anthocyanins present in *C. mas* fruit is given in Figure 2.

## 4 AA

Flavonoids from the fruits of *C. mas* may possess beneficial health effects, especially by acting as powerful antioxidants [65].

In most of the studies, the AA of the fruits was determined by the 2,2-diphenyl-1-picrylhydrazyl (DPPH) free radical method. According to the results of Milenković-Andjelković et al. [29], 100 ml of cornelian cherry fruit extract scavenged 94–109 mg of DPPH radicals. Tural and Koca [42] observed that the methanol extracts of cornelian cherry fruit showed EC50 (mg·ml^−1^) values (DPPH reduction) as 0.52. Dragović-Uzelac et al. [66] found that DPPH values in two different species of cornelian cherry were as high as 33.41 and 39.89 mmol Trolox equivalent·kg^−1^ FW.

The final value of AA is influenced by the method of detection used, as different techniques may yield varying results. Additionally, AA is also cultivar and genotype dependent. Klymenko et al. [23] assessed AA in 20 cultivars of cornelian cherry cultivated in Ukraine by DPPH, ABTS (ABTS assay is a colorimetric assay in which the ABTS radical suffers a color decrease in the presence of antioxidants), and fluorescence recovery after photobleaching (FRAP) assays. The values AA (μmol Trolox·g^−1^) determined by DPPH method showed values ranging from 5.94 (“Kozerog”) to 16.56 (“Kostia”), in ABTS method varied from 13.560 (“Koralovyj Marka”) to 33.96 (“Semen”), in FRAP method ranged from 8.45 (“Koralovyj”) to 22.49 (“Kostia”). Kucharska et al. [8] studied the AA of Polish breeding varieties and determined the highest activity in the variety “Dublany” (20.72 μmol Trolox·g^−1^) and the lowest in the variety “Juliusz” (10.85 μmol Trolox·g^−1^).

The color of fruit is closely related to its chemical composition, which determines also its antioxidant properties. The cultivar “Uholok” with almost black fruits showed the highest antioxidant potential, while cultivars with pink fruits displayed the lowest potential [2]. The antioxidant capacity is genotype dependent, as demonstrated in the study of Cosmulescu et al. [24] (2.39 and 2.71 mmol Trolox 100 g^−1^ FW, respectively).

A study conducted by Yilmaz et al. [26] showed considerable variability in FRAP values, with a minimum of 73 and a maximum of 114 μmol of AA equivalent g^−1^ DW. Pantelidis et al. [67] confirmed that the FRAP value is approximately 84 μmol AA·g^−1^ DW.

The bioactive compounds in fruit exhibit a synergistic effect, which is reflected in the relationship between AA and chemical composition of fruit [17].

Hassanpour et al. [68] stated that the DPPH radical scavenging efficiency depends on the total concentration of polyphenolic compounds, total flavonoids, and AA (Pearson coefficients: 0.54, 0.60, and 0.47).

Radical scavenging activity (DPPH) significantly correlated with total phenolic content (*R* ^2^ = 0.9832) [29]. TPC positively correlated with FRAP (*r* = 0.845, *F* < 0.05) and DPPH assays (*r* = 0.706, *p* < 0.05). A very weak correlation (*r* = 0.102) was found between TPC and ABTS [23]. A positive correlation between AA and polyphenols has been demonstrated in studies by Ersoy et al. [69] and Milenković-Andjelković et al. [29]. *C. mas* fruit extracts exhibited strong AA, which correlated positively with the total phenolic content and phenolic compounds such as anthocyanins and flavonols, but did not correlate with the iridoid content. In *C. mas* cultivars, AA was dependent on fruit color; thus, the cultivar Uholok with black fruits showed the highest antioxidant potential [2].

According to Cetkovská et al. [27], the AA determined by electron paramagnetic resonance and DPPH radical scavenging assay ranged from 29.5 to 67.2%. They studied different cultivars grown in the conditions of the Czech Republic.

The antioxidant behavior significantly depends on the cultivar of the species and its genotype [13].

AA is also affected by processing and storage conditions. Storage of fruit at low temperature (freezing), the AA of fruit, liqueurs prepared from frozen fruit had a higher antioxidant capacity and dry weight than liqueurs produced in the traditional way [70]. The antioxidant capacity values of stored CM fruits were temperature dependent. Moldovan et al. [71] observed a 10% decrease in the antioxidant capacity of CM fruits stored at 2°C for 60 days. Higher losses were observed when stored at 75°C, with an average 29% reduction in total vitamin C capacity after 10 days.

## 5 Biological effect of fruit

The fruit of the cornelian cherry has been valued for medical properties [2]. Among frequently reported health-promoting effects, antidiabetic, antiatherogenic, anti-inflammatory, anticancer, and neuroprotective effects can be mentioned [9,18,39,72]. The health-promoting activity of the fruit summarized in Table 2 is provided by polyphenolic compounds which displayed the highest AA [13,73].

**Table 2:** Main biological effects of *C. mas* L. fruits

Biological effect	Mechanism of action	Source
Anti-inflammatory	Suppression of IL-1β and IL-13	Moldovan et al. [41]
	Reduction of IL-6 and TNF-α concentrations	Sozański et al. [74], Wójciak et al. [75]
	Increase in TNF-α and IL-1β secretion	Czerwińska et al. [76]
	Immunomodulatory activity – regulations of the Th17/Treg	Szandruk-Bender et al. [77]
	Decrease in C-reactive protein	Aryaeian et al. [78]
	Decrease in the release of NO, IL-12, and TNF	Crisan et al. [79]
Antimicrobial	Supression of the propagation of *Bacillus*, *Escherichia coli*, *Staphyloccocus aureus*, *Pseudomonas aeruginosa*	Bayram and Ozturkcanm [3], Aurori et al. [7]
	Inhibition of *Staphylococcus aureus*, *Candida* sp., and *Aspergillus fumigatus* (methanolic extract)	Krisch et al. [80], Krzyściak et al. [81], Turker et al. [82]
Metabolic and antidiabetic effect	Inhibitory effect on α-amylase and α-glucosidase	Shishehbor et al. [83], Blagojević et al. [11], Okan et al. [43], Sip et al. [84]
	Decrease in leptin and increase in adiponectin reduction of triglycerides	Danielewski et al. [85]
	Regulating insulin sensitivity in adipocytes	Małodobra et al. [14]
	Stimulation of the secretion of insulin by pancreatic β cells	Jayaprakasam [86]
	Inhibition of pancreatic lipase activity by pelargonidin 3-*O*-galactoside	Świerczewska et al. [64]
Cardioprotective effect	Reduction in MMP-1, IL-6, and NOX mRNA expression in the aorta	Danielewski et al. [87]
	Decrease in VCAM-1, ICAM-1, PON-1, MCP-1, and PCT serum level	
	Decrease in triglycerides (*p* < 0.001)	Moldovan et al. [88]
	C-reactive protein	
	Antiplatelet effects and reduction of platelet hyper-reactivity	Lietava et al. [89]
Anticancer effect	Inhibition of breast adenocarcinoma cell line growth	Odzakovic et al. [90]
	Cytotoxic, antiproliferative effect	Šavikin et al. [91]; Rezaei et al. [92]; Hosseini et al. [93]; Tiptiri-Kourpeti et al. [94]; Lewandowski et al. [95]
	Downregulation of Bcl-2	Ji et al. [96]

Abbreviations: TNF-α, tumor necrosis factor; IL-1β IL-13, IL-12, IL-6, interleukins; NO, nitric oxide; MMP-1, metalloproteinase-1; NOX, nitric oxidases; VCAM-1, vascular cell adhesion molecule-1; ICAM-1, intercellular adhesion molecule-1; PON-1, paraoxonase-1; MCP-1, monocyte chemotactic protein-1; PCT, procalcitonin; Bcl-2, gene – apoptosis regulator.

### 5.1 Anti-inflammatory effect

Extracts from cornelian cherry have been found to be potential anti-inflammatory agents in both *in vitro* and *in vivo* studies, even in situations where cornelian cherries have been processed into functional foods or traditional dishes [39,89].

The anti-inflammatory activity of cornelian cherry fruit extract was demonstrated in various models of Wister rats. The ability of the extract to suppress the levels of interleukin (IL)-1β and IL-13 [37,41] or to reduce the concentration of IL-6 and tumor necrosis factor (TNF)-α, [74] was studied. Czerwińska et al. [76] observed the effect *C. mas* fruit extract on reactive oxygen species generation in human neutrophils as well as cytokines secretion both in neutrophils (TNF-α, IL-8, IL-1β) and in human colon adenocarcinoma cell line Caco-2 (IL-8). The results of the experiment showed that the aqueous-ethanolic extract of *C. mas* fruit had a tendency to increase TNF-α and IL-1β secretion. On the other hand, the modulatory activity of *C. mas* extracts was observed in the case of IL-8 secretion in Caco-2 cells. Similarly, a study by Wójciak et al. [75] indicated that cornelian cherry extract suppressed the production of IL-6, IL-8, and TNF-α in human skin keratinocytes and fibroblasts. Loganoic acid and cornuside were identified as the primary compounds responsible for this effect.

Szandruk-Bender et al. [77] studied the effect of cornelian cherry (*C. mas* L.) extract, rich in iridoids and polyphenols, at two doses (20 or 100 mg kg^−1^ FW) on the crucial factors for Th17/Treg-cell differentiation in the course of experimental colitis. They demonstrated a beneficial effect of cornelian cherry extract on experimental colitis which they explained by immunomodulatory activity – regulations of the Th17/Treg developmental pathway molecules due to the high polyphenolic content in the extract. Th17/Treg promotes the development of chronic inflammatory disorders and induces autoimmune diseases.

Regular consumption of *C. mas* extract resulted in a decrease in highly sensitive C-reactive protein in postmenopausal women [78].

Crisan et al. [79] assessed that the effect of polyphenolic extract from cornelian cherry fruit on psoriasis displayed anti-inflammatory activity. They described the mechanism of action of silver and gold nanoparticles (GNPs) complexed with *C. mas* (Ag-NPs-CM, Au-NPs-CM) in psoriasis at the cellular and molecular levels. The experimental results showed that incubation of pro-inflammatory macrophages with the nanoparticles significantly reduced the release of NO (nitric oxide), IL-12, and TNF. NPs-CM (nanoparticles) appear to repress NF-B (nuclear factor-B) activation in macrophages, inhibiting the production of pro-inflammatory factors with a causal role in psoriasis.

Another clinical study on the influence of CM (*C. mas*) fruit extract on ameliorating lipid profile and vascular inflammation in 40 dyslipidemic children and adolescents aged 9–16 years was conducted by Asgary et al. [97]. The results demonstrated a trend toward improvement in lipid profile and vascular inflammation after adding CM to the daily diet of dyslipidemic children and adolescents.

### 5.2 Antimicrobial activity

The fruits are well known as an important antimicrobial agent displaying significant activity against numerous bacteria and fungi including *Bacillus*, *Escherichia coli*, *Staphylococcus aureus*, and *Pseudomonas aeruginosa*. The majority of studies have identified methanol, aqueous, and ethanol extracts of fruits as potent antimicrobial agents [3]. Methanol extract of CM seeds inhibited *S. aureus*, fungi *Candida*, and *Aspergillus fumigatus*, whereas ethanol extract of CM seeds inhibited *S. aureus* and *Candida albicans*. Methanol extract of CM fruits displayed a moderate effect on *S. aureus*, *E. coli*, and *P. aeruginosa*, while ethanol extract of CM fruits displayed a moderate effect on *E. coli* and *P. aeruginosa* [80,81].

The 50°C ethanol extract of CM fruits has a strong antibacterial activity against *S. aureus*, *Staphylococcus epidermidis*, and *Streptococcus pyogenes* [82].

Significant antimicrobial activity against Gram-positive, followed by Gram-negative strains and yeasts, was observed for all tested extracts [29].

Aurori et al. [7] used the ethanolic extract *of C. mas* fruit to determine the antimicrobial and cytoprotective effects *in vitro* on renal cells stressed with gentamicin. The antimicrobial activity was investigated by agar well diffusion and broth microdilution methods, with excellent results for *P. aeruginosa.*

The aim of the study by Savas et al. [98] was to determine the antimicrobial effects of cornelian cherry marmalade “Garagurt.” The antimicrobial activity of the sample was evaluated by disc diffusion method at a minimum inhibitory concentration against *Listeria monocytogenes*, *Bacillus cereus*, *S. aureus*, *E. coli*, *E. coli* O157:H7, *Salmonella typhimurium*, *Pseudomonas fluorescens*, and *Yersinia enterocolitica*. The results of the study showed that the sample provided antimicrobial and AA (195 ± 6.35 mg GAE·100 g^−1^) in aqueous methanol extract of Garagurt. This product could be, therefore, used for its antimicrobial effect to increase the shelf life of various foods [98].

### 5.3 Metabolic and antidiabetic effects

The fruit of cornelian cherry can be widely used in prevention of metabolic disorders, including diabetes [99].

Shishehbor et al. [83] studied the inhibitory effects of hydroalcoholic extracts from cornelian cherry (*C. mas* L.) fruit extracts against pancreatic α-amylase and intestinal α-glucosidase. The results indicated that cornelian cherry was an effective inhibitor of α-glucosidase with the IC50 values of 6.87 mg·ml^−1^. The fruit extract was recommended for use in controlling postprandial hyperglycemia.

Capcarova et al. [100] studied the effect of orally administered *C. mas* fruit on the development of diabetes mellitus symptoms in ZDF rats (the Zucker fatty rat). In the experiment, male ZDF rats (fa/fa) and their age-matched non-diabetic lean controls (fa/+) were utilized. Male ZDF rats were administered two doses (500 and 1,000 mg kg^−1^ BW) for 10 weeks. The control group received only distilled water. The control and experimental rats were fed with normal chow – KKZ-P/M (complete feed mixture for rats and mouse, reg. no 6147, Dobra Voda, Slovak Republic) on an *ad libitum* basis. They found a significant decrease in glucose levels after oral administration of *C. mas* at a dose of 1,000 mg kg^−1^ BW in the prediabetic state of the animals (up to the 7th week of the experiment) and a significant reduction in water intake in both *C. mas* groups compared to the control. It was concluded that a higher dose of cornelian cherry could be beneficial and useful in preventing diabetic symptoms consumed regularly in young animals [100].

Cornelian cherry (*C. mas* L.) has been recommended as an alternative treatment for type 2 diabetes mellitus (T2DM). The effect of a polyphenolic extract from the pulp and pit of cornelian cherry was studied in ZDF (the Zucker fatty rat) rats. ZDF rats received cornelian cherry in three different doses (1,500, 2,000 mg·kg^−1^ BW of the pulp, and 250 mg·kg^−1^ BW of the stone) for 4 months. Blood glucose, insulin, AA, and compounds in cornelian cherry were monitored and measured. The experimental results revealed that cornelian cherry pit significantly reduced the affected blood glucose level in ZDF rats compared to the control group. The content of total polyphenols and phenolic acids was significantly higher in the pit of cornelian cherry than in the pulp. At the same time, high concentrations of anthocyanins were determined in the pulp [36].

Fruits of *C. mas* L. are valued not only for their hypoglycemic effects but also in the treatment of a very important secondary complication of T2DM – diabetic bone disease. Omelka et al. [101] studied the effect of *C. mas* pulp on lipid profile and bone quality parameters in Zucker diabetic rats (ZDF). They discovered that cornelian cherry pulp could be used as a potential therapeutic agent to alleviate T2DM of reduced bone quality and impaired bone health. In addition, the lipid-lowering properties of this fruit have also been identified.

Cornelian cherry fruit extract positively influences metabolism – lowering leptin levels and increasing adiponectin concentrations and lowering triglycerides (TGs), as opposed to total cholesterol (TC) and LDL in rats fed a high-fat diet [85].

Most recent literature emphasizes antidiabetic properties of *C. mas* fruit and products [76,102]*. C. mas* L. is one of the most valuable fruit plants with hypoglycemic potential. One proven mechanism of effect is based on inhibition of digestive enzymes or glucose uptake [64]. The fruits display an inhibitory effect on exocrine enzymes responsible for breaking down complex carbohydrates (α-amylase and α-glucosidase) into easily digestible simple sugars [11,43]. Sip et al. [84] offered a new approach to diabetes therapy based on α-glucosidase inhibition and the antioxidant effect resulting from the activity of the plant extract used, combined with the prebiotic effect of inulin. A study by Małodobra et al. [14] proved that cornelian cherry fruit extract can reverse insulin resistance by expression of genes involved in the transmission of the insulin signal or regulating insulin sensitivity in adipocytes.

7-*O*-Galloyl-d-sedoheptulose decreased the levels of advanced glycation end products-related proteins as well as some inflammation-related protein expressions in hepatic tissues [103,104]. In addition to reducing BW and hepatic lipids in rats, anthocyanins of cornelian cherry correctly alleviate the course of diabetes [86]. Moreover, anthocyanins stimulate β cells in the rodent pancreas to secrete insulin and improve glucose tolerance and insulin resistance [105]. Furthermore, anthocyanins isolated from CM fruits at a dose of 500 mg·kg^−1^ BW per day for 8 weeks reduced serum total TG and TC, resulting in a 24% reduction in BW [86].

The high AA of cornelian cherry fruit extract can contribute to the prevention of changes in blood cell structure that can occur in diabetes. Ethanolic extract of red and yellow CM fruits (20 mg·kg^−1^) administered by intraperitoneal injection for 14 days reduced FBG (area under the glycemic curve index) and increased glucose intolerance [47].

As mentioned, the fruits of *C. mas* possess anti-inflammatory activity, which is essential in the treatment of diabetes [106].

Świerczewska et al. [64] demonstrated that pelargonidin 3-*O*-galactoside isolated from CM fruits (7.5 mg·ml^−1^) significantly inhibited pancreatic lipase activity by 28.3 ± 1.5%. They recommended the fruits as a food for the prevention of hyperlipidemia-related diseases.

### 5.4 Skin

*C. mas* L. fruit extracts may be an effective strategy to prevent skin cell damage caused by free radicals. The aqueous glycerin extract of dried cornelian cherry has proven to be a rich source of antioxidant compounds, such as phenols and flavonoids, and is capable of scavenging free radicals in a dose-dependent manner, as demonstrated by Wójciak et al. [75]. Novel nanoparticle-based biomaterials carrying polyphenol-rich extracts (*C. mas*) have recently shown promising anti-inflammatory activity in psoriasis [75].

### 5.5 Cardioprotective effect

Sozański et al. [107] reported the cardioprotective effect of loganoic acid and anthocyanins isolated from the fruits of *C. mas* L. on diet-induced atherosclerosis in rabbits. Danielewski et al. [87] applied a *C. mas* L. fruit extract rich in iridoids and anthocyanins (at doses of 10 or 50 mg kg^−1^ BW) to a rabbit model of a cholesterol-rich diet. The application resulted in a significant reduction in the mRNA expression of metalloproteinase-1, IL-6, and NOX (oxidases) in the aorta and a decrease in serum levels of vascular cell adhesion molecule-1, intercellular adhesion molecule-1, paraoxonase-1, monocyte chemotactic protein-1, and procalcitonin. The best results were obtained at a dose of 50 mg kg^−1^ BW. They concluded that cornelian cherry fruit extract may be useful in atherogenesis-related cardiovascular diseases such as atherosclerosis or metabolic syndrome.

Moldovan et al. [88] applied GNPs functionalized with bioactive compounds extracted from *C. mas* L. fruits (AuNPsCM) in an experimental model of a high-fat diet and evaluated the effects of the extract on the aortic wall, but also in serum, compared to different administration of *C. mas* fruits (CM). The results of the study showed that AuNPsCM exhibited better effects on lipid peroxidation (*p* < 0.01) and TNF-α (*p* < 0.001) in aortic homogenates compared to the natural extract (CM). CM and AuNPsCM exhibited hypolipidemic TG (*p* < 0.001) and C-reactive protein (CM, *p* < 0.01; AuNPsCM, *p* < 0.001) lowering effects.

The fruits of *C. mas* L. show high antioxidant potential, reduce inflammatory markers, have a significant effect on the lipid spectrum (comparable to statins), reduce glycemia, and increase insulin levels. The polyphenols of cherry cornelian exhibit both direct antiplatelet effects and a reduction in platelet hyperreactivity. Clinical studies on the administration of polyphenol extract of *C. mas* L. fruits have shown clinically relevant reductions in TC and low-density lipoprotein, triacylglycerols, lipoproteins, improvement in inflammatory activity, and improvement in insulin secretion. *C. mas* fruit extract also reduced inflammatory markers – the treatment showed beneficial effects on lipid spectrum (comparable to statins), reduction in glycemia, and increase in insulin. According to the results of studies by Lietava et al. [89], fruit consumption is recommended as an effective tool for the treatment of atherosclerosis.

### 5.6 Anticancer activity

Consumption of wild cornelian cherry extract can have a significant anti-cancer potential, which has been suggested in samples from Macedonia. The first CC3 sample from Drinic with the highest anthocyanin content (1.40 mg CyGE·g^−1^ FW) inhibited free radicals (IC50DPPH = 262.19 mg·ml^−1^; IC50ABTS = 76.78 mg·ml^−1^; IC50OH center dot = 102.31 mg·ml^−1^) and inhibited the growth of a breast adenocarcinoma cell line (IC50MCF-7 = 1.37 mg·ml^−1^). The second sample CC4 from Drvar showed the highest content of total polyphenols (55.92 mg GAE·g^−1^ DW) and vitamin C (88.74 mg·g^−1^ FW) and significantly inhibited the growth of cervical epithelioid carcinoma (IC50HeLa = 0.62 mg·ml^−1^) and lung adenocarcinoma cell line (IC50A549 = 0.48 mg·ml^−1^) [90].

Recently, the cytotoxic, antiproliferative, and anticancer properties of fruits have received attention from various researchers [91,92,93].

Perde-Schrepler et al. [108] studied two different cell lines, normal keratinocytes and A431, epidermoid carcinoma GNPs (spherical between 2 and 24 nm in size) with the addition of CM extract. The experimental results recommend GNPs-CM for further testing and possible dermatological applications.

Tiptiri-Kourpeti et al. [94] demonstrated that cornelian cherry has significant AA against the free radical 2,2-diphenyl-1-picrylhydrazyl (DPPH) and moderate antiproliferative potential against four human cancer cell lines and one mouse cell line: MCF-7 mammary adenocarcinoma, HepG2 hepatocellular carcinoma, and Caco2, HT-29 colon adenocarcinomas, as well as CT26 murine colon carcinoma. Cell viability decreased by 40–50% after incubation with the highest concentration of juice.

Cornelian cherry fruits are characterized by high antioxidant capacity and antiproliferative activity on colon cancer cells (HT29) [11].

Lewandowski et al. [95] studied the cytotoxic effect of fruit extract isolated from *C. mas* L. – red-stained fruit (cultivar “Podolski”) and yellow-stained *C. mas* L. (“Yantarnyi” and “Flava”) on two melanoma cell lines (A375 and MeWo). Cytotoxicity was analyzed using sulforhodamine B assay and a colorimetric assay for assessing cell metabolic activity methods (sulforhodamine B assay and 3-(4,5-dimethylthiazol-2-yl)-2,5-diphenyltetrazolium bromide). The results showed that both extracts from *C. mas* L. induced cytotoxicity in A375 and MeWo cell lines.

Ji et al. [96] determined the antitumor potential of cornelian fruit extract on major regulatory genes in renal carcinogenesis. They observed down-regulation of Bcl-2 (apoptosis regulator) expression as an anti-apoptotic gene by 4.34-fold due to the addition of the extract. In addition, messenger RNA expression of the Her2 oncogene was inhibited by a concentration of 250 μg ml^−1^ similar to 10-fold CME.

Aside from the health-promoting effect of polyphenols, it is also important to consider their side effects, particularly when consumed in supplement form rather than in their natural state. Using supplement form represents an uncontrolled method of treating diseases, as it often contains high doses of pure polyphenols [109].

Except for medicinal values, the cornelian cherry is valuable because it is undemanding in cultivation and able to grow in an extreme environment. Due to early ripeness, it represents a valuable source of vitamin C and another bioactive compound. It has an important landscape-forming as well as an aesthetic, rehabilitation, isolation, and cultural function [110].

## 6 Conclusions

Recently, *C. mas* has been considered a minor, forgotten, and underutilized fruit species in the modern world. However, the fruit of *C. mas* has been widely used in folk medicine and for the production of various drinks, syrups, gels, jams and compotes, liqueurs, and wines. Polyphenols, including flavonols, anthocyanins, flavan-3-ols, and phenolic acids, along with iridoids, largely contribute to its AA and the associated health-promoting effects such as anti-inflammatory, antidiabetic, cardioprotective, and anti-cancer properties. Research has shown that the content of polyphenolic compounds varies significantly and depends on factors such as the origin (cultivation territory), genotype, method of sample extraction, detection methodology, and processing techniques. Nowadays, there has been a growing interest in cultivating new varieties of *C. mas* that are rich in biologically active compounds, including polyphenols, that can have a wide range of potential applications in medicine and as functional foods. Further studies are needed to explore the mechanism of action of polyphenolic compounds of fruit as well as studies focused on their bioavailability.
